# The Pattern of Use of Cosmetics and Awareness of Cosmetovigilance Among Medical Students in Puducherry: A Cross-Sectional Study

**DOI:** 10.7759/cureus.78335

**Published:** 2025-02-01

**Authors:** P Abiramy, Balagurumoorthy Maharani, Mohanan Saritha, Prakash Mathiyalagen

**Affiliations:** 1 Pharmacology, Indira Gandhi Medical College and Research Institute, Puducherry, IND; 2 Dermatology, Indira Gandhi Medical College and Research Institute, Puducherry, IND; 3 Community Medicine, Indira Gandhi Medical College and Research Institute, Puducherry, IND

**Keywords:** adverse event, allergy, health care providers, knowledge, questionnaire

## Abstract

Background: With the increasing use of cosmetics, potential adverse effects such as allergic reactions and skin irritation warrant investigation. This study aims to assess cosmetic usage patterns, associated adverse effects, and awareness of cosmetovigilance among medical students. Despite the widespread use of cosmetics in India, region-specific data on adverse effects and cosmetovigilance awareness in Puducherry are limited. Given medical students' future roles in patient education, assessing their awareness is crucial. The study was conducted among medical students, who are expected to have a foundational understanding of the adverse effects of cosmetics due to their training in pharmacology and dermatology. However, their actual awareness of cosmetovigilance remains unclear, warranting this investigation.

Methods: This cross-sectional, questionnaire-based study was conducted among all MBBS students studying in a tertiary care teaching hospital in Puducherry. The questionnaire was reviewed by dermatologists and pharmacologists for face and content validity, and internal consistency was tested using Cronbach’s alpha, which was found to be reliable (0.73). The pretested, content-validated questionnaire, which included demographic details of the participants, the nature and type of cosmetics used, adverse reactions (if any) experienced by them, and awareness of cosmetovigilance, was shared along with the informed consent form and participant information sheet via Google Forms. Responses were obtained from the participants.

Results: Of the 600 eligible students, 246 participated, resulting in a response rate of 41%. The most commonly used cosmetics among participants were skin cleansing products, hair and scalp cleansing products, and tooth care products, respectively. Among the respondents, 71.4% (n=178) were aware that cosmetic products may cause adverse events, and 49.59% (n=122) had experienced adverse events following the use of cosmetics. Only 27.6% (n=68) of students were aware of the term cosmetovigilance. The majority became aware of the term through social media (17.5%, n=43). Among all participants, 42.2% (n=105) expressed willingness to report adverse drug reactions (ADRs) associated with the use of cosmetics in the future by filling out an ADR reporting form.

Conclusion: Given the low awareness of cosmetovigilance among medical students, integrating it into the medical curriculum is essential to enhance future healthcare professionals' role in patient safety. Future studies should focus on assessing reporting barriers, awareness gaps among dermatologists, and differences in knowledge between medical students and the general public.

## Introduction

Cosmetics are defined under India’s Drugs and Cosmetics Act (1940) and its 1945 rules as “any article intended to be rubbed, poured, sprinkled, or sprayed on, or introduced into, or otherwise applied to, the human body or any part thereof for cleansing, beautifying, promoting attractiveness, or altering the appearance, and any article intended for use as a component of a cosmetic.” The manufacturing and licensing of cosmetics in India are regulated by this Act [1]. The Drug Controller General of India (DCGI) oversees the import of cosmetics [2]. The Ministry of Health and Family Welfare (MOHFW) introduced the Cosmetics Rules (2020) to codify and update regulations related to the import, manufacturing, labeling, sale, and distribution of cosmetics in India [3]. The use of cosmetics dates back to ancient civilizations, including the Indus Valley Civilization, though safety regulations have evolved significantly in recent decades [4].

Public awareness of cosmetic products has increased significantly, with personal grooming gaining more importance in modern society. Most people, regardless of gender or age, are highly conscious of their appearance, leading to a 20% annual growth in the Indian cosmetic market [5]. The Indian cosmetic product market is expected to grow at a Compound Annual Growth Rate (CAGR) of 4.23% by 2025 [5]. A wide range of cosmetic products is available in the market, categorized as skin care products, hair and scalp products, nail and cuticle products, and oral hygiene products [6]. Cosmetics contain chemical compounds derived from natural or synthetic sources and are classified into organic, herbal, ayurvedic, and synthetic formulations [7].

COSMOS is an international body certifying organic and natural cosmetics and their ingredients [8]. Despite regulatory oversight, the Indian market faces challenges with counterfeit cosmetics, including misbranded and mislabeled products, as well as those containing unapproved ingredients [9]. Products with names or packaging resembling another cosmetic or manufacturer, and those with misleading or fictitious information that may deceive consumers, are labeled as spurious cosmetics [9]. While cosmetic products are not intended to harm human health, their use is often associated with adverse reactions ranging from mild to serious effects, potentially leading to morbidity or mortality [10,11]. Furthermore, in some cases, adverse reactions to cosmetics are very mild and may go unnoticed due to the absence of a proper reporting system.

Hence, the cosmetovigilance program was implemented with the objective of “activities related to the collection, evaluation, and monitoring of spontaneous reports of undesirable events observed during or after normal or reasonably foreseeable use of a cosmetic product” [12]. It serves as a major mechanism for obtaining safety information on cosmetic products and their ingredients [13]. Additionally, it aids in identifying and eliminating hazardous ingredients in cosmetics [12]. The increased use of cosmetics among adults and the potential hazards associated with their use make it necessary to study the pattern of cosmetic use, the adverse effects associated with cosmetics, and awareness of cosmetovigilance among the public.

In today’s world, where the utilization of cosmetics is increasing, data on the pattern of cosmetic use and awareness of cosmetovigilance remain limited in India and nearly nonexistent in the Union Territory of Puducherry. Hence, this study was conducted among medical students, as they have exposure to pharmacology and dermatology and are an ideal group to assess awareness of cosmetovigilance.

Aim

The objective of this study was to assess the usage patterns of cosmetics, the incidence of associated adverse effects, and the level of cosmetovigilance awareness among medical students in Puducherry. The primary objective was to evaluate the usage patterns of cosmetics and the prevalence of associated adverse effects among medical students. The secondary objective was to determine the level of cosmetovigilance awareness among medical students in Puducherry.

## Materials and methods

This cross-sectional, questionnaire-based study was conducted among MBBS students (first-year to interns) aged 18 years and above at a tertiary care teaching hospital in Puducherry. The study was conducted over two months (October 1, 2023, to November 30, 2023) following approval from the Institutional Ethics Committee (No. 492/IEC-38/PP-3/2023, dated 05.09.2023) and the relevant college authorities.

Sample size calculation

The sample size was determined using the formula: N = 4pq/L², where p (prevalence of adverse events from cosmetics) was taken as 12.2% based on previous studies [14], q = 100 - p = 87.8%, and L (allowable error) = 5%. The estimated sample size was 171. To account for potential non-responses and incomplete data, the final target sample size was set at 200.

Study tool and data collection

A pretested, content-validated questionnaire (circulated among five dermatologists and five pharmacologists to assess content validity and face validity; internal consistency was measured using Cronbach’s alpha and found to be reliable (0.73)) was used to collect data on participant demographics, cosmetic usage patterns, adverse reactions (if any), and awareness of cosmetovigilance. The validated questionnaire, along with the informed consent form and participant information sheet, was distributed via Google Forms [10,15]. The Google Form was shared with participants via institutional social media groups (WhatsApp). Students were instructed to complete all questions and select appropriate responses where applicable. The questionnaire was designed to be completed in less than 10 minutes to minimize disruption to the participants' routine. Weekly reminders were sent via student social media groups to encourage participation until the target sample size was achieved.

Statistical analysis

The Google Form responses were exported to Google Sheets and analyzed using IBM SPSS Statistics for Windows, Version 16 (Released 2007; IBM Corp., Armonk, New York). Descriptive statistics were presented as mean ± standard deviation (SD) for numerical variables and as percentages and proportions for categorical variables. Statistical tests were selected based on the nature and distribution of variables. The chi-square test and Fisher's exact test were used for categorical variables, while the independent t-test was applied for numerical variables after assessing normality. A p-value < 0.05 was considered statistically significant. Following data collection, participants were provided with information on cosmetovigilance, ADR reporting, and its importance in enhancing awareness as part of the post-research responsibility of the researcher.

## Results

A total of 246 MBBS students from a tertiary care teaching hospital participated in the study by completing the questionnaire, resulting in a response rate of 41%. The mean age of participants was 20.53 ± 1.70 years. The majority of respondents were female (n = 148, 60.2%). Among the respondents, 34.6% (n = 85) were first-year students, 32.5% (n = 80) were second-year students, 4.9% (n = 12) were third-year students, 24% (n = 59) were final-year students, and 4.1% (n = 10) were interns (Table 1).

**Table 1:** Demographic details of study participants

S. No.	Variable	Type	Number (percentage)
1	Sex	Male	98 (39.8)
		Female	148 (60.2)
2	Year in course	First year	85 (34.6)
		Second year	80 (32.5)
		Third year	12 (4.9)
		Final year	59 (24)
		Intern	10 (4.1)
3	Per capita income	Less than INR 500/month	178 (72.4)
		INR 501-1000/month	45 (18.3)
		INR 1001-5000/month	21 (8.5)
		More than INR 5000/month	2 (0.8)

The most commonly used cosmetics were skin products (93%), followed by hair and scalp products (87%) and oral hygiene products (84.8%). Nail and cuticle products were the least commonly used (17.4%). The use of skin products, hair and scalp products, and oral hygiene products among participants is depicted in Table 2.

**Table 2:** Patterns of cosmetic use among participants *P-value <0.05 is considered significant.

Variable	Type	Male, n (%)	Female, n (%)	P-value (chi-square)
Skin product type	All types	88 (35.7)	141 (57.3)	0.097
	Skin care products	42 (17.07)	105 (42.7)	<0.001*
	Correction of body odor products	41 (16.6)	57 (23.17)	0.602
	Body hair removal products	10 (4.06)	51 (20.7)	<0.001*
	Skin cleansing products	73 (29.7)	120 (48.8)	0.218
	Fairness products	14 (5.7)	33 (13.4)	0.118
	Makeup products	4 (1.6)	65 (26.4)	<0.001*
	Products to reduce suntan	29 (11.8)	74 (30.08)	0.001*
	Body hair bleach	2 (0.8)	3 (1.2)	0.994
	Body hair care products	6 (2.4)	12 (4.8)	0.558
	Perfumes	61 (24.7)	87 (35.4)	0.587
	Lip care products	16 (6.5)	80 (32.5)	<0.001*
	Pre- and after-shave products	33 (13.4)	31 (12.6)	0.026*
Hair product type	All types	91 (36.99)	123 (50)	0.026*
	Cleansing products	82 (33.3)	116 (47.15)	0.305
	Styling products	11 (4.47)	18 (7.3)	0.823
	Colouring products	1 (0.4)	5 (2.03)	0.241
Nail products type	All types	8 (3.2)	35 (14.2)	0.002*
	Nail varnish and removal products	2 (0.8)	30 (12.2)	<0.001*
	Nail glue and remover	1 (0.4)	5 (2.03)	0.310
	Nail care products	2 (0.8)	6 (2.4)	0.497
Oral hygiene type	All type	90 (36.5)	119 (48.3)	0.014*
	Tooth care products	90 (36.5)	118 (47.9)	0.010*
	Tooth whiteners	11 (4.5)	6 (2.4)	0.030*
	Mouth wash	34 (13.8)	41 (16.6)	0.244
Nature of cosmetic	Herbal	10 (4.06)	26 (10.5)	0.110
	Ayurvedic	6 (2.4)	14 (5.6)	0.348
	Organic	10 (4.06)	33 (13.4)	0.014*
	Natural	18 (7.3)	62 (25.2)	<0.001*
	Synthetic	52 (21.1)	95 (38.6)	0.081
Expenditure per month on cosmetics (INR)	<500	74 (30.1)	104 (43.9)	0.682
	501-1000	17 (6.9)	28 (11.3)	
	1001-5000	6 (2.4)	15 (6.1)	
	>5000	1 (0.4)	1 (0.4)	

The most commonly used skin care product, hair and scalp product, and oral hygiene product among participants were skin cleansing products, hair and scalp cleansing products, and tooth care products, respectively. Most participants used oral hygiene and skin care products daily, hair care products weekly, and nail and cuticle products rarely. In addition to synthetic cosmetics (n = 147, 59.7%), participants also used natural (n = 80, 32.5%), organic (n = 43, 17.5%), herbal (n = 36, 14.6%), and Ayurvedic products (n = 20, 8%) (Table 2). There was a significant difference in the use of skin care products, hair and scalp products, nail and cuticle products, oral hygiene products, hair removal products, makeup products, products to reduce suntan, lip care products, products used for shaving, tooth care products and whiteners, organic cosmetics, and natural cosmetics between genders. On average, 72.4% (n = 178) of participants spent less than INR 500 on cosmetics per month.

**Figure 1:**
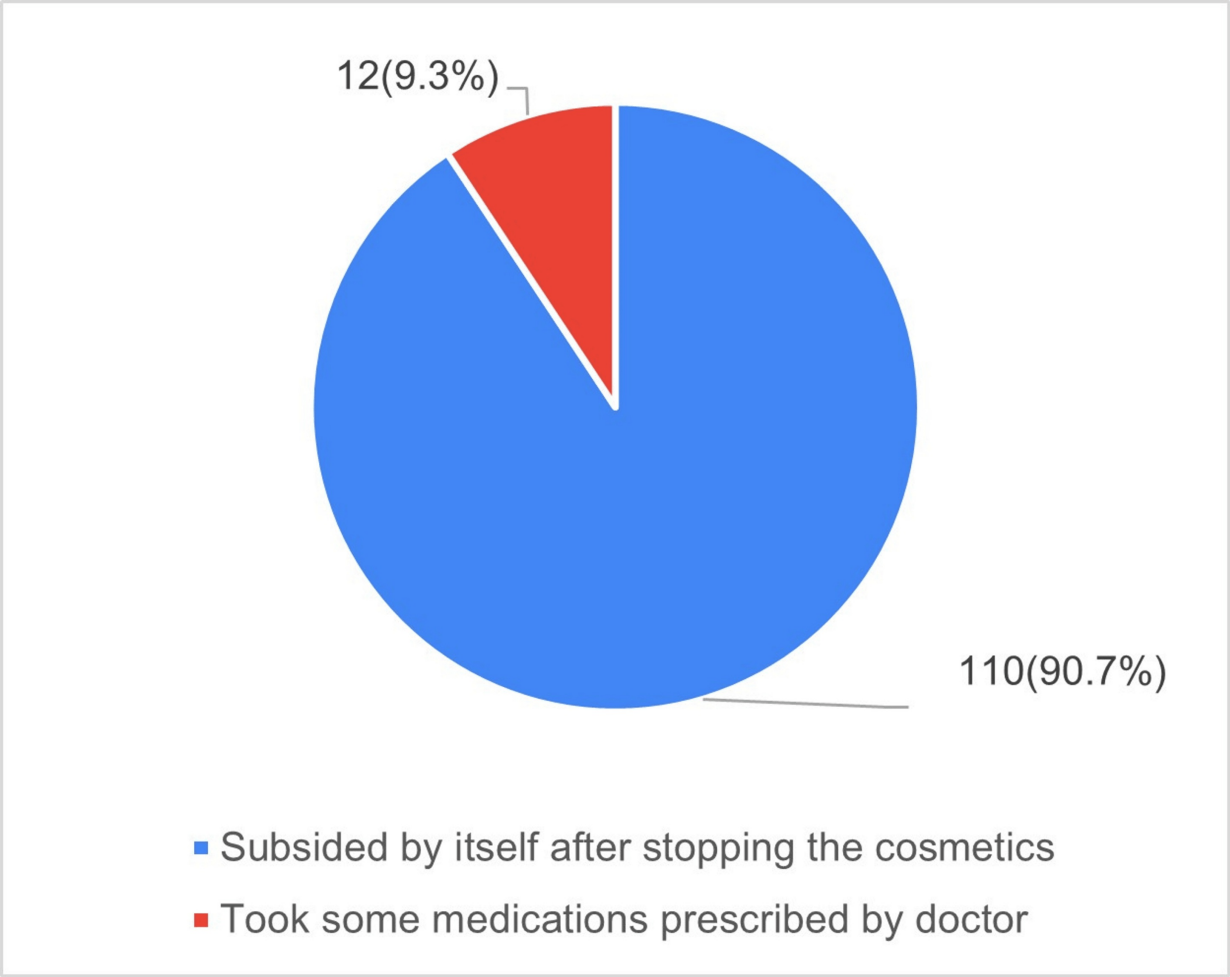
Management of ADR following use of cosmetics ADR: adverse drug reaction.

Among respondents, 71.4% (n = 176) were aware that cosmetics may cause adverse events, and 49.5% (n = 122) had experienced adverse reactions. Among participants who experienced ADRs (n = 122), 42.7% (n = 105) had mild reactions, and 6.8% (n = 17) had moderate reactions. Table 3 presents information on adverse reactions associated with cosmetic use, and Figure 1 presents the management of ADRs following the use of cosmetics.

**Table 3:** AR of cosmetics *P value <0.05 is considered significant. AR: adverse reaction, PPD: para-phenylenediamine, ADR: adverse drug reaction, CME: continuing medical education.

Variable	Type	Male, n (%)	Female, n (%)	P-value
Aware cosmetics cause AR	-	58 (23.5)	118 (47.9)	<0.001*
Experienced AR	-	39 (15.8)	83 (33.7)	0.001*
Aware AR can be reported	-	45 (18.3)	89 (36.2)	0.028*
Severity of reaction	Mild	33 (13.4)	72 (29.3)	0.042*
	Moderate	6 (2.4)	11 (4.4)	0.041*
Additives causing AR	Silicones	17 (6.9)	44 (17.8)	0.145
	Sulfates	24 (9.7)	60 (24.4)	0.036*
	Parabens	20 (8.13)	57 (23.17)	0.022*
	PPD	37 (15)	62 (25.2)	0.203
	Fragrances	16 (6.5)	43 (17.4)	0.165
	All of the above	13 (5.2)	27 (10.9)	0.331
Reporting AR to	Pharma firm	25 (10.2)	48 (19.5)	0.859
	Medical officer	20 (8.1)	33 (13.4)	0.457
	Pharmacist/store	20 (8.13)	29 (11.7)	0.178
	Food/drug authority	16 (6.5)	29 (11.7)	0.731
	ADR monitoring center	26 (10.5)	55 (22.3)	0.653
Awareness of cosmetovigilance	Yes	22 (8.9)	46 (18.7)	0.760
	No	23 (9.3)	43 (17.4)	
Source of information	Social media	15 (6.09)	28 (11.4)	0.152
	Friends	9 (3.6)	14 (5.7)	0.393
	Textbooks	15 (6)	21 (8.5)	0.700
	Lectures	10 (4.06)	22 (8.9)	0.048*
	CME/workshops	7 (2.8)	11 (4.47)	0.782
Willingness to report	Yes	32 (13)	73 (29.2)	0.147
	No	13 (5.2)	16 (6.5)	

Most of the ADRs were caused by synthetic cosmetics (35.4%), followed by herbal, Ayurvedic, and organic products (21% each), while the lowest incidence of ADRs was reported with the use of natural products (13.8%). There was a significant difference between genders in awareness that cosmetics can cause adverse reactions, with female students experiencing significantly more adverse reactions than males. Female students experienced significantly higher rates of hair fall, new onset of acne, skin irritation, split ends, skin itching, loss of natural hair texture, facial itching, stinging sensation, erythema, abnormal peeling, skin discoloration, changes in skin tone, exacerbation of acne, scalp itching, reactions involving the lips, and fragile skin, among others (Table 4).

**Table 4:** ADR symptoms experienced by participants *P-value <0.05 is considered significant. ADR: adverse drug reaction.

ADR	Male, n (%)	Female, n (%)	P-value
Hair fall	18 (14.8)	50 (41)	<0.001*
New incidence of acne	14 (11.5)	41 (33.6)	<0.001*
Skin irritation	7 (5.7)	39 (32)	<0.001*
Split ends	8 (6.6)	38 (31.1)	<0.001*
Skin itching	3 (2.5)	30 (24.6)	<0.001*
Loss of natural hair texture	8 (6.6)	27 (22.1)	<0.001*
Dry and dehydrated hair strands	16 (13.1)	26 (21.3)	0.089
Aggravation of dandruff	15 (12.3)	26 (21.3)	0.059
Itching of face	2 (1.6)	23 (18.9)	<0.001*
Stinging sensation	1 (0.8)	21 (17.2)	<0.001*
Erythema	2 (1.6)	18 (14.8)	<0.001*
Exacerbation of acne	7 (5.7)	18 (14.8)	0.020*
Scalp itching	8 (6.6)	18 (14.8)	0.038*
Eye irritation	3 (2.5)	13 (10.7)	0.009*
Abnormal peeling	1 (0.8)	11 (9)	0.003*
Discoloration of skin	1 (0.8)	11 (9)	0.003*
Skin color change	1 (0.8)	11 (9)	0.003*
Photosensitivity	3 (2.5)	9 (7.4)	0.075
Reaction involving lips	0	7 (5.7)	0.0142*
Stretch marks	4 (3.3)	6 (4.9)	0.5184
Fragile skin	0	6 (4.9)	0.029*
Hair root infection	1 (0.8)	4 (3.3)	0.369
Discoloration of hair	3 (2.5)	3 (2.5)	>0.9
Blisters and oozing	1 (0.8)	2 (1.6)	>0.9
Paronychia	0	1 (0.8)	>0.9
Others	0	1 (0.8)	>0.9
Nausea	1 (0.8)	0	>0.9
Dyspnoea	0	0	>0.9

There was significantly greater awareness among female students than male students that adverse reactions to cosmetics can be reported. Only 27.6% (n = 68) of students were aware of the term cosmetovigilance. The majority became aware of the term through social media (17.5%, n = 43). There was no significant difference between genders in awareness of the term "cosmetovigilance." The majority (32.8%, n = 81) believed that ADRs could be reported to an ADR monitoring center by filling out an ADR reporting form. Other methods of reporting selected by respondents are depicted in Table 3. Among all participants, 42.2% (n = 105) expressed willingness to report ADRs related to cosmetics in the future by submitting an ADR reporting form.

## Discussion

The word "cosmetic" is derived from the Greek word "kosmētikos," meaning "to adorn" [16]. According to the G.S.R 426(E) Gazette notification, cosmetics are categorized into four major groups: skin products, hair and scalp products, nail and cuticle products, and products for oral hygiene [9]. Each category is further divided into multiple subcategories. Among the broad categories, the most commonly used cosmetics among our study respondents were skin products (93%), while the least used were nail and cuticle products (17.4%). This finding was similar to a study by Lucca et al., which revealed that makeup products (24.56%) were the most commonly used cosmetics, categorized as skin care products [15]. In another study from South India, talcum powder and compact powder were the most frequently used products, followed by facial soaps and shampoo [17]. Our study found that the use of skin care products, body hair removal products, makeup products, products to reduce suntan, and lip care products among female participants was significantly higher (P = <0.001) than among male participants. A similar pattern was observed in the usage of nail products. The pattern of cosmetic use in our study differed from previous studies [18].

The use of organic (17.5%) and natural (32.5%) cosmetic products was significantly higher among female participants than male participants, as per self-reported classification in our study. A study from Sri Lanka found that only 40% of young women had heard about green cosmetics [19], indicating that awareness and usage of green cosmetics were higher among our participants. Nearly 27.6% of participants spent more than INR 500 on cosmetics, which is considerably higher than the 3.35% reported in a study conducted in South Kerala among medical students [18], possibly due to evolving consumer trends and increased product availability.

Cosmetovigilance was introduced in the late 1990s as a public health initiative aimed at monitoring and ensuring the safety of cosmetic products [9]. To assess the causality of ADRs related to cosmetic products, the AFSSAPS (French Health Products Safety Agency) method and the definition by Colipa are used. Our study sought to assess only the pattern of adverse drug reactions (ADRs) experienced by participants using cosmetic products and their awareness of the concept of cosmetovigilance among medical students [20]. A study on 800 women found that 41.1% of working women and 35.1% of nonworking women experienced adverse effects [17]. Another study from Ethiopia found that 61% of cosmetic users developed adverse effects [21]. Various studies conducted in different parts of the world have shown that cosmetics can cause ADRs, with the incidence of ADRs from cosmetic usage ranging from 12.2% to 61% [14,15,17,22-25].

In our study, 49.59% of respondents experienced adverse effects, yet 45.5% of participants were unaware of adverse reaction reporting or the cosmetovigilance system (27.6%). Only 42.2% of participants were willing to report ADRs associated with the use of cosmetics in the future by filling out an ADR reporting form. This is likely because most of the population considers cosmetics to be harmless [15]. In India, adverse reactions have been reported with commonly used kajal and kumkum [9,26]. These findings establish that cosmetovigilance is the need of the hour [9]. In a study conducted by Rathi et al., the most commonly encountered adverse reactions to cosmetics included dermatitis, tissue damage, infection, discoloration, bleeding, nervousness, respiratory system reactions, vomiting, diarrhea, urogenital reactions, and even flammability-induced death [6]. In our study, the most common adverse effects were hair fall, new onset of acne, skin irritation, and split ends, while systemic adverse effects such as nausea were the least reported. Similarly, systemic adverse effects like nausea and headache were rarely reported in a previous study [17].

Cosmetovigilance is the process of collecting, analyzing, and assessing adverse reactions occurring in consumers using cosmetics to identify potential health risks, thereby ensuring enhanced consumer safety [6]. The term was first used in French literature in 1997 [12]. Due to a lack of a well-organized reporting system in India, undesirable reactions to cosmetic products often go unreported [11]. One study found that India contributed only 3.82% of all published literature on cosmetic adverse effects [11]. The authors noted that it is imperative for India to establish a robust reporting system for ADRs associated with cosmetic products to prevent spurious and substandard cosmetics from dominating the market and to protect consumer health.

Our study has a few limitations. Since participants self-reported via Google Forms, responses may be prone to error. The study is also subject to recall bias, as individuals who experienced adverse effects are more likely to remember details regarding cosmetic use than those who do not recall the nature of the cosmetics they used. Another limitation is that causality assessment was not conducted for adverse effects related to cosmetics, making these reports subject to bias. Additionally, since the surveyed population consisted of medical students with exposure to healthcare practices, it may be challenging to extrapolate these findings to the general population.

## Conclusions

Cosmetovigilance is essential for promoting safe cosmetic use, particularly in an increasingly competitive market. In India, greater efforts are needed to enhance cosmetovigilance awareness among healthcare providers and the public through education and improved reporting systems. Further population-based studies are necessary to evaluate adverse effects associated with cosmetic products and to assess awareness of cosmetovigilance among healthcare providers and the general public. These findings will help guide targeted awareness initiatives to ensure safer cosmetic use.
